# Tryptophan Hydroxylase-2-Mediated Serotonin Biosynthesis Suppresses Cell Reprogramming into Pluripotent State

**DOI:** 10.3390/ijms24054862

**Published:** 2023-03-02

**Authors:** Sergey A. Sinenko, Andrey A. Kuzmin, Elena V. Skvortsova, Sergey V. Ponomartsev, Evgeniya V. Efimova, Michael Bader, Natalia Alenina, Alexey N. Tomilin

**Affiliations:** 1 Institute of Cytology, Russian Academy of Sciences, Tikhoretsky Ave. 4, 194064 St. Petersburg, Russia; 2 Institute of Translational Biomedicine, St-Petersburg State University, 7–9 Universitetskaya Emb, 199034 St. Petersburg, Russia,; 3 Max-Delbruck Center for Molecular Medicine, 13125 Berlin-Buch, Germany

**Keywords:** serotonin, 5-HT, pluripotency, reprogramming, induced pluripotent stem cells (iPSCs), tryptophan hydroxylase 1 and 2 (TPH1 and TPH2)

## Abstract

The monoamine neurotransmitter serotonin (5-hydroxytryptamine, 5-HT) has important functions both in the neural system and during embryonic development in mammals. In this study, we set out to investigate whether and how endogenous serotonin affects reprogramming to pluripotency. As serotonin is synthesized from tryptophan by the rate limiting enzymes tryptophan hydroxylase-1 and -2 (TPH1 and TPH2), we have assessed the reprogramming of TPH1- and/or TPH2-deficient mouse embryonic fibroblasts (MEFs) to induced pluripotent stem cells (iPSCs). The reprogramming of the double mutant MEFs showed a dramatic increase in the efficiency of iPSC generation. In contrast, ectopic expression of TPH2 alone or in conjunction with TPH1 reverted the rate of reprogramming of the double mutant MEFs to the wild-type level and besides, TPH2 overexpression significantly suppressed reprogramming of wild-type MEFs. Our data thus suggest a negative role of serotonin biosynthesis in the reprogramming of somatic cells to a pluripotent state.

## 1. Introduction

The monoamine serotonin (5-hydroxytryptamine, 5-HT) plays an important role in a broad range of physiological processes, working either as an autacoid (in the periphery) or as a neurotransmitter (in the central nervous system). The biosynthesis of serotonin starts with conversion of the essential amino acid tryptophan (Trp) to 5-hydroxytryptophan (5-HTP), by the rate-limiting enzymes tryptophan hydroxylases (TPHs). In the second step, 5-HTP is decarboxylated to serotonin by aromatic amino acid decarboxylase (AADC). Two different genes, *Tph1* and *Tph2,* encoding the highly homologous TPH1 and TPH2 enzymes, exist in vertebrates [1], serving as the basis for two independent serotonin systems [2]. TPH2 is specifically expressed in the serotonergic neurons of raphe nuclei of the brainstem [1,3], and in myenteric neurons in the gut [4]. TPH1 is responsible for peripheral serotonin synthesis in the enterochromaffin cells of the gut and pineal gland. Serotonin is secreted from the gut to the blood stream and is accumulated by platelets [5,6]. Due to the fact that serotonin does not cross the blood–brain barrier, the two serotonin biosynthesis systems are totally independent in their regulation and function. Serotonin binds and signals through 15 known specific receptors of seven families, 5-HT_1_ through 5-HT_7_ [7,8]. Both in the peripheral organs and in the brain, the extracellular serotonin levels are regulated by the serotonin transporter (SERT, Slc6a4). Serotonin is taken up by tissues, then either degraded or retained for future release [9].

Serotonin can also act while being covalently bound to glutamine (Gln) residues on proteins by transglutaminases (TG), drastically modifying the activity of these targeted proteins. This mechanism, called “serotonylation”, was discovered to play a role in platelets [1,10]. Small G-proteins and extracellular matrix proteins are modified by serotonylation, which is involved in the regulation of insulin secretion and the proliferation of vascular smooth muscle cells [10,11,12]. Recently, it has been shown that serotonylation of Gln occurs in position 5 (Q5ser) of histone H3 and histone H3 tri-methyl lysine 4 (H3K4me3)-marked nucleosomes, resulting in the presence of the combinatorial H3K4me3Q5ser modification that is enriched in the euchromatin of neurons. This histone modification is required for proper neuronal differentiation and correlates with permissive gene expression [13].

Neurons producing serotonin provide widespread innervation throughout the central nervous system already at early stages of development. Mice deficient in brain serotonin have abnormalities in a broad range of autonomous functions, such as the regulation of sleep/wakefulness, thermoregulation, cardiovascular function, respiration, appetite, as well as in social and maternal behavior [14,15,16,17]. It has been shown that the levels of serotonin biosynthesis and signaling during mouse development have multifaceted roles in supporting adult hippocampal neurogenesis [16,18,19].

Mounting evidence suggests that serotonin is not only a neurotransmitter but also plays a role in the early development and morphogenesis of both invertebrates and vertebrates. Serotonin was shown to be pivotal for the first embryonic cell divisions in several classes of invertebrates [20,21]. In vertebrates, TPH2, SERT, 5-HT2, and 5-HT7 receptor expression was detected from the zygote to blastocyst stages and in cultured embryonic stem cells (ESCs) [22,23,24,25], pointing to some functions of serotonin in these cells. At early stages of embryogenesis, serotonin is thought to modulate cell proliferation, migration, cell shape, and cell–cell interactions. Later during embryogenesis, serotonin is essential for left–right symmetry establishment in vertebrate embryos, as well as for the survival, migration, and differentiation of mouse cranial neural crest (NC) cells [26,27,28,29,30,31,32,33]. TPH2-dependent serotonin is involved in the development of NC-derived tissues as a morphogenetic factor [33,34]. Right after birth, brain serotonin seems to be essential for normal growth, because TPH2-deficient (*Tph2^−/−^*) mice, which completely lack the monoamine in the brain but have unchanged levels in the periphery, exhibit pre-weaning growth retardation [14,35].

In 2006, a breakthrough in the stem cell research field was made by Takahashi and Yamanaka, who showed, for the first time, that pluripotent state could be induced in somatic cells via forced co-expression of 4 transcription factors – Oct4, Sox2, Klf4, and cMyc [36]. This discovery allowed the derivation of autologous induced pluripotent stem cells (iPSCs) from somatic cells, thus getting around the issue of immune incompatibility, as well as around the ethical issue of human ESC derivation [37,38]. There had been no evidence for a role of serotonin in the reprogramming of somatic cells to iPSCs, however, the observation that the 5-HT_3_ receptor agonist, 2-methylserotonin, could replace Oct4 during this process, spurred some interest [39]. Nevertheless, neither the 5-HT receptors, nor signaling mechanisms, nor serotonergic drugs have been studied in the context of cellular reprogramming. In this paper, we provide compelling evidence for a significant role of the serotonin-producing enzyme TPH2 in this process.

## 2. Results

### 2.1. Loss of Serotonin Synthesis Improves the Efficiency of iPSC Generation

**Figure 1:**
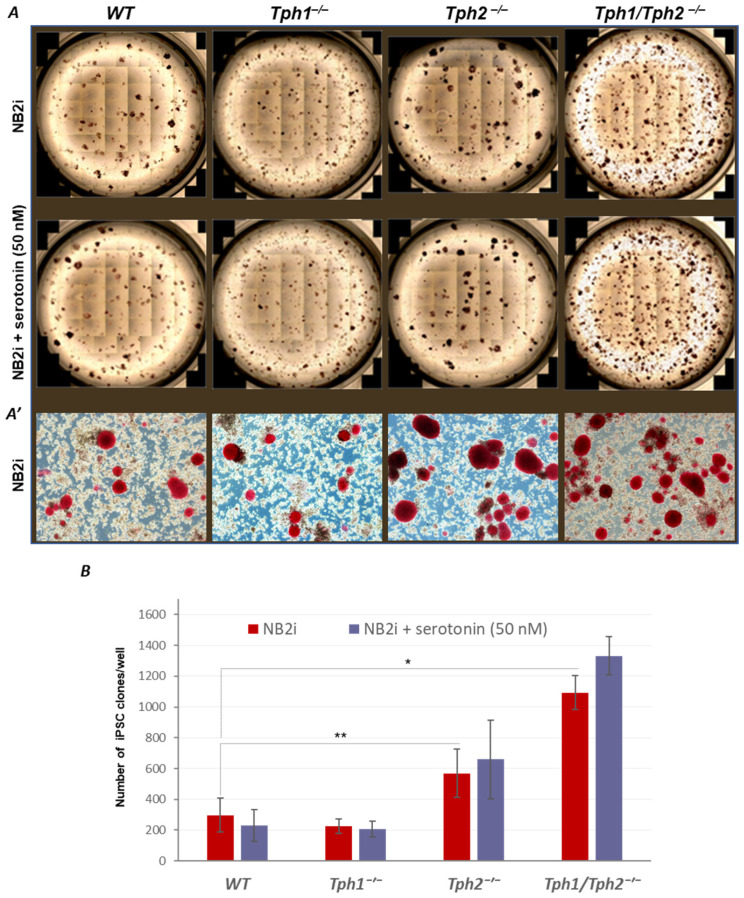
Loss of serotonin (5-HT) biosynthesis leads to a robust increase of the efficiency of OKSM-mediated MEF reprogramming to iPSCs regardless of serotonin presence in the medium. MEFs of the indicated genotypes were reprogrammed using an inducible rtTA/OKSM polycistronic vector under serum-free N2B27-2i (NB2i) conditions. (**A**) Color photographs of 3-cm wells containing alkaline phosphatase (AP)-positive iPSC clones (red staining) generated without (upper panel) or in the presence of serotonin at 50 nM (lower panel). (**A’**) View of the AP-positive clones from corresponding wells at a higher (10-fold) magnification. (**B**) Counts of AP-positive clones generated during the reprogramming experiments presented in A. The reprogramming of the *Tph1/Tph2^−^*^/*−*^ and *Tph2^−/−^* MEFs was about four and two times as efficient as that of wild-type (WT) MEFs (red bars), respectively, and was not affected by 50 nM serotonin presence in the medium (blue bars). *Y*-axis represent counts of the AP-positive iPSC clones ± standard deviations (SD); *n* = 4, * *p* < 0.0005, ** *p* < 0.05; Student’s *t*-test.

To find out if serotonin biosynthesis has a role in the reprogramming of somatic cells to pluripotency, we have made use of mice harboring either *Tph1^−/−^* or *Tph2^−/−^* genes [1,2,14], as well as of more recently developed double knockout mouse, *Tph1/Tph2^−/−^* [40,41,42]. Mouse embryonic fibroblasts (MEFs) from these mutants and WT mice of the same genetic background were compared for their capacity of being reprogrammed into iPSCs in N2B27 + 2i media, using a rtTA-inducible lentiviral polycistronic OKSM vector. We have found that loss of both the TPH1 and TPH2 enzymes leads to a significant (up to 5 times) increase in the number of generated iPSC clones, compared to that of WT MEFs (Figure 1A,B). As opposed to that, *Tph1^−/−^* MEFs showed similar to their WT counterparts reprogramming rate. Yet, *Tph2^−/−^* MEFs displayed about 2-time increase of reprogramming efficiency, compared to the WT counterparts (Figure 1A,B). Importantly, the presence of serotonin in culture media at concentration of 50 nM during all stages of the cell reprogramming did not affect its efficiency (Figure 1B, blue bars), suggesting that extracellular serotonin does not interfere with the outcome of reprogramming.

Although it is likely that the lack of TPH2 is primarily responsible for the enhanced rate of iPSCs generation, the difference in the reprogramming efficiency between *Tph1/Tph2^−/−^* and *Tph2^−/−^* MEFs suggests a possible engagement of TPH1 into the reprogramming process in the absence of TPH2 activity. Indeed, using pan-TPH antibodies, we observed that in contrast to *Tph1/Tph2^−/−^* and *Tph2^−/−^* iPSCs, which expectedly did not express any TPH, the total TPH protein was dramatically upregulated in *Tph1^−/−^* iPSCs, reflecting the overexpression of TPH2 in the context of TPH1 deficiency (Supplementary Figure S1).

Further analysis of the generated TPH mutant iPSCs demonstrated expression of the pluripotency markers alkaline phosphatase (AP) and Nanog at levels comparable to those of WT cells (Figure 1A,A’, Supplementary Figure S4). The analysis also showed the capacity of mutant cells to differentiate into derivatives of the three embryonic germ layers within teratomas (Supplementary Figure S2). All together, these data suggest that serotonin biosynthesis inhibits the reprogramming into a pluripotent state.

### 2.2. Ectopic Tph2 Expression Suppresses iPSCs Generation and Restores the Rate of Reprogramming of Tph1/Tph2^−/−^ MEFs to the WT Level

**Figure 2:**
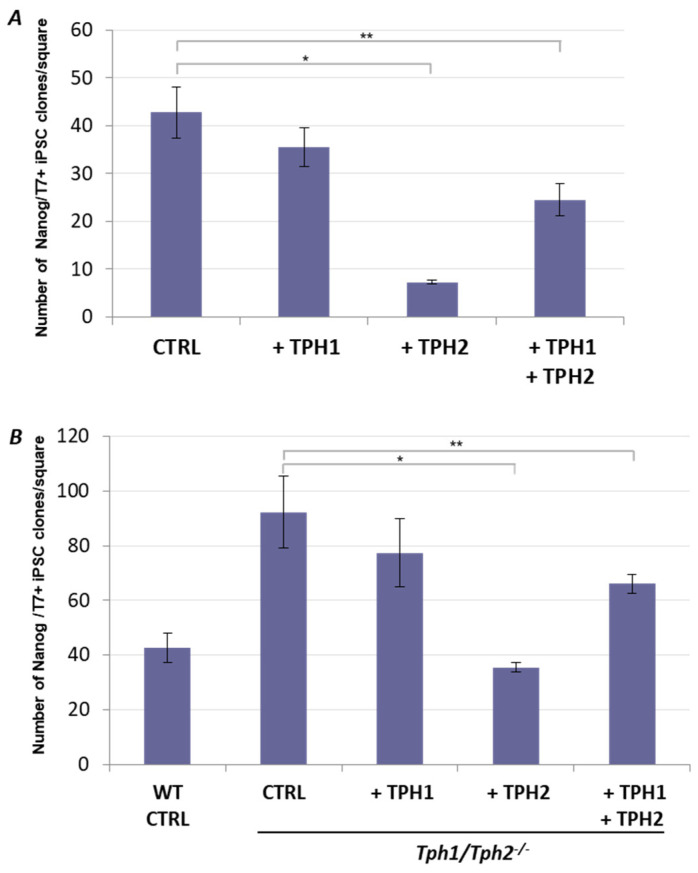
TPH2 suppresses cell reprogramming to pluripotent state. (**A**) Ectopic expression of TPH1 and TPH2 during reprogramming of WT MEFs. iPSC clones were revealed by co-immunostaining for Nanog and T7 epitope which tagged the exogenous TPH proteins. Ectopic expression of TPH2 alone or in conjuncture with TPH1 significantly suppresses efficiency of iPSC generation by OKSM lentiviruses. (**B**) Ectopic expression of TPH2 during reprogramming of *Tph1/Tph2^−^*^/*−*^ MEFs completely reverted its efficiency to the WT level, while its co-expression with TPH1 had a lesser effect. Overexpression of TPH1 alone did not significantly affect the reprogramming efficiency of *Tph1/Tph2^−^*^/*−*^ MEFs. Equal numbers of the TPH-encoding lentiviruses were supplemented during the reprogramming (A and B), and if one or both of them were omitted, the total lentivirus amounts were adjusted with the T7 tag-encoding mock lentivirus (CTRL). *Y*-axes represent counts of the double-positive iPSC clones ± SD; *n* = 4 (technical replicates); * *p* < 0.0005, ** *p* < 0.005 (Student’s *t*-test).

Next, we set out to investigate the effect of increased serotonin production, achieved via the excess of TPH enzymes during the process of reprogramming into iPSCs. To this end, TPH1, TPH2, or both proteins, were overexpressed as T7-tagged fusion proteins, during the OKSM-mediated cell reprogramming of the WT MEFs, with the help of lentiviral vectors. Consistent with the above results on TPH-deficient MEFs, TPH2 overexpression in WT cells dramatically (up to 8 times) suppressed the reprogramming efficiency, as compared with the empty lentiviral vector Figure 2A. Simultaneous overexpression of TPH1 and TPH2 also significantly suppressed the efficiency of cell reprogramming, albeit to a lesser extent than TPH2 alone. However, overexpression of TPH1 alone had little effect (Figure 2A, Supplementary Figure S3). These results confirm the above conclusions that TPH2 activity is primarily involved in serotonin biosynthesis during the reprogramming, and that the produced serotonin acts as a suppressor of this process.

To further support the revealed function of serotonin biosynthesis during cell reprogramming, we performed rescue experiments, in which the ectopic TPH1 and TPH2 were provided to the *Tph1/Tph2^−^*^/*−*^ MEFs during reprogramming. As expected, continuous ectopic expression of TPH2 in *Tph1/Tph2^−^*^/*−*^ MEFs during their reprogramming attenuated the positive effect of the TPH-deficiency, while co-expression of both TPH1 and TPH2 also suppressed the reprogramming efficiency of *Tph1/Tph2^−/−^* MEFs, albeit to a lesser extent (Figure 2B and Supplementary Figure S4). However, similar to the above experiment, overexpression of TPH1 alone was not sufficient to revert the *Tph1/Tph2^−^*^/*−*^ phenotype. These results further substantiate the idea that TPH2-mediated serotonin synthesis is involved in the negative regulation of cellular reprogramming to pluripotency.

### 2.3. Suppression of Serotonylation by ZDON Does Not Enhance the Reprogramming Efficiency

**Figure 3:**
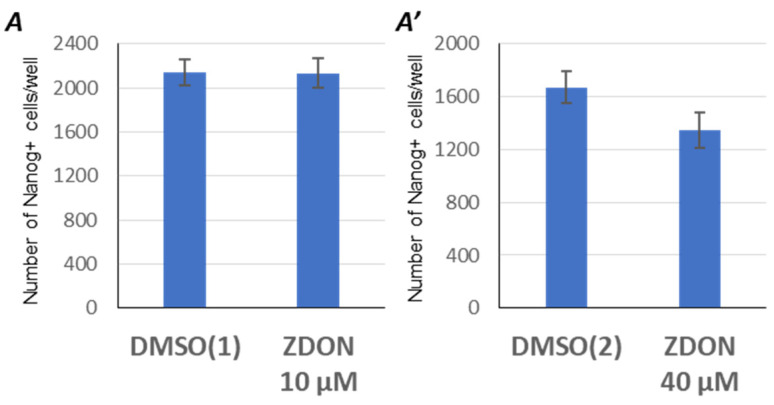
Serotonylation is not involved in the reprogramming to pluripotency. Inhibiting serotonylation with 10 μM (**A**) or 40 μM ZDON (**A’**) does not enhance the reprogramming efficiency of wild-type MEFs, as compared to cells treated with corresponding concentrations of vehicle, designated as DMSO (1) and (2). *Y*-axes represent counts of the Nanog-positive iPSC clones ± SD; *n* = 3 (technical replicates); *p* < 0.005.

We next hypothesized that one possible pathway of intracellular serotonin action could involve the regulation of potential target proteins via serotonin-mediated post-translational modification, referred to as serotonylation [10,12]. To investigate if the serotonylation is involved in cell reprogramming, we made use of a potent, cell-permeable, and irreversible inhibitor of transglutaminase 2—6-diazo-5-oxo-norleucine tetrapeptide (ZDON) [43]. However, treatment with this inhibitor during the whole reprogramming period did not improve its outcome: on average, 2133±133 and 2178±119 of Nanog-positive iPSC-clones were generated in the presence of 10 μM ZDON or its vehicle (DMSO), respectively (Figure 3A,A’, Supplementary Figure S5). Furthermore, an increase of ZDON concentration to 40 µM slightly reduced the reprogramming efficiency. Thus, inhibition of serotonylation during the reprogramming does not recapitulate the *Tph1/Tph2^−^*^/*−*^ phenotype, suggesting that intracellular serotonin during the reprogramming functions via a pathway other than serotonylation.

**Figure 4:**
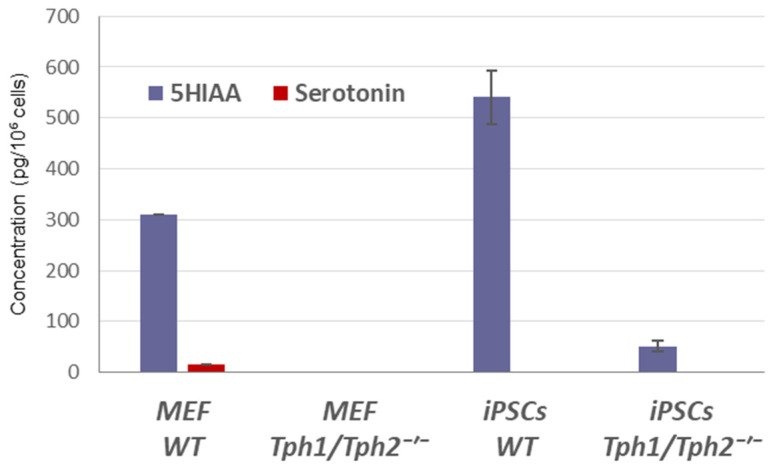
Intracellular serotonin metabolite 5-HIAA levels depend on TPH activity. Serotonin and 5-HIAA levels in cell lysates from wild-type (WT) and *Tph1/Tph2^−/−^* MEFs, as well as from iPSC counterparts, cultured in 10% FBS-containing MEF medium or serum-free N2B27-2i medium, respectively. *Y*-axis represent concentration (pg/10^6^ cells) ±SD; *n* = 2 (technical replicates), *p* < 0.001.

Fetal bovine serum (FBS)-containing culture medium, used both for MEF maintenance and during the first three days of reprogramming, is a known source of serotonin. Indeed, HPLC analysis detected serotonin at concentrations of 32, 50, and 82 ng/mL in media containing 5%, 10%, and 15% FBS, respectively, whereas serum-free N2B27 and Opti-MEM media were essentially free of serotonin (Supplementary Figure S6). This observation raises the possibility that FBS-derived serotonin might be taken up and accumulated in MEFs and iPSC precursors, interfering with the outcome of reprogramming. However, we observed only trace amounts (or none) of intracellular serotonin in lysates of WT MEFs and *Tph1/Tph2*^−/−^ MEFs, respectively (Figure 3B). In contrast, significant amounts of hydroxyindoleacetic acid (5-HIAA), the main metabolite of serotonin, were readily detected in cell lysates of WT MEFs and iPSCs, but not in the *Tph1/Tph2*^−/−^ counterparts, regardless of the presence of FBS in the medium (Figure 4). Immunostaining of WT MEFs and iPSCs with pan-TPH antibodies revealed expression of this enzymes in rare MEFs, and a high expression—in iPSCs (Supplementary Figure S7). These results suggest intracellular rather than extracellular origin of serotonin in MEFs, iPSCs, and likely, in iPSC precursors during the OKSM-mediated reprogramming. Furthermore, it appears that this serotonin is immediately metabolized to 5-HIAA by monoamine oxidases, probably due to the inability of these cells to package serotonin into secretory vesicles [41].

## 3. Discussion

Serotonin signaling is involved in the regulation of many different developmental processes in metazoans [14,44,45]. Synthesis of serotonin is solely dependent on the rate limiting TPH enzymes. Two different genes encoding TPH1 and TPH2 enzymes are highly conserved through metazoans and function in peripheral and neural tissues, respectively [41,46,47]. We found that knockout of the both *Tph* genes in MEFs dramatically increases the efficiency of their reprogramming into iPSCs. However, knockout of a single gene produced either mild (*Tph2)* or no effect (*Tph1*) on reprogramming. The lack of an effect of TPH1 loss on cell reprogramming is probably due to compensatory upregulation of TPH2. On the other hand, overexpression of TPH2, but not of TPH1, suppressed reprogramming of WT MEFs and attenuated the *Tph1/Tph2^−/−^* reprogramming phenotype. This lack of functional redundancy may suggest that TPH1 is regulated differently in given cellular context, becoming unstable or inhibited when overexpressed. Another enzyme, phenylalanine hydroxylase (PAH), belonging (together with TH and TPH) to the family of pterin-dependent hydroxylases that convert Trp to 5-HTP [40], might also be involved, generating additional complexity in regulation and compensating TPH loss. Further studies are required to clarify the revealed functional differences between the *Tph* genes in the context of cellular reprogramming. Nevertheless, loss of intracellular serotonin biosynthesis in *Tph1/Tph2^−^*^/*−*^ MEFs sufficiently enhances the cell reprogramming, suggesting that serotonin production suppresses the generation of iPSCs. It is well known that serotonin is maintained at very low levels in most tissues and cells [48,49,50]. In our experiments, no serotonin *per se* was detected in WT iPSCs, while it was readily detected in WT MEFs. We assume that serotonin levels may be elevated in MEF-derived iPSC-precursors during certain stages of the reprogramming process, which requires further analysis.

While the mechanism of the intracellular serotonin function in the cell reprogramming remains to be elucidated, one can hypothesize that it can operate through interaction with metabolic fluxes and, particularly, with mitochondrial function. In support of this idea, it has been shown that serotonin mediates the mitochondrial stress response in the neurons of *C. elegans* [51]. In cortical neurons, serotonin signaling along with the master regulators of mitochondrial biogenesis—sirtuin-1 and peroxisome proliferator-activated receptor gamma coactivator 1-α (PGC-1α)—positively regulate generation of mitochondria and enhance mitochondrial function [52]. It has also been shown that TPH2 expression inversely correlates with expression of genes related to mitochondrial functions, indicating that TPH2 could be involved in protecting neurons against mitochondrial dysfunction [53]. In addition, within intrapulmonary arteries, serotonin induces O_2_^−^ production via 5-HT receptors [54], while in breast cancer cells it enhances mitochondrial biogenesis [55]. Several 5-HT receptors have been detected on the mitochondrial membrane and found to participate in the regulation of mitochondrial activities, including ROS generation. It is well known that ROS have important signaling functions in different developmental processes [56,57]. It has also been established that optimal mitochondrial ROS levels are important for efficient reprogramming to iPSCs [58,59]. Therefore, the possibility that intracellular serotonin signaling affects the reprogramming via mitochondrial ROS production deserves further investigation.

Tryptophan, in addition to being the sole precursor of serotonin, is a well-known and important precursor of kynurenine (Kyn), vitamin B3, as well as of an essential redox regulator, nicotinamide adenine dinucleotide (NAD). It has also been shown that Trp metabolism plays a key role in promoting the proliferation of human ESCs and iPSCs through activation of N-formylkynurenine (NFK)/N-formylanthranilic acid (NFAA) and suppression of Kyn/NAD metabolic pathways [53,60,61]. The presence of Kyn in culture medium is a biomarker of undifferentiated ESCs, but not of differentiated cells [62]. To date, it has been shown that Trp-based NAD synthesis is mainly restricted to hepatocytes and does not take place in fibroblasts or pluripotent stem cells. Each of these Trp-based metabolic branches may act at a certain stage of the reprogramming. Clearly, the exact molecular mechanism of serotonin and Trp-dependent metabolic pathways in the regulation of the reprogramming to pluripotency requires further investigation.

To date, a large number of small molecule compounds, including metabolic regulators and antioxidants, have been found to promote reprogramming to pluripotency. The discovery of these molecules improves the efficiency of somatic cell reprogramming and helps to understand the molecular mechanism of this process [63,64,65]. Some studies have shown that small molecules acting on metabolic regulation can directly alter the levels of DNA or histone modification, thereby affecting the reprogramming outcome [65]. We have identified a novel (suppressive) function of serotonin biosynthesis during MEF reprogramming to iPSCs. This study raises important questions regarding the specific functions of TPH2-mediated serotonin biosynthesis and Trp metabolism in the regulation of reprogramming to pluripotency. It also indicates that these pathways may operate in certain cellular and developmental processes. From a practical point of view, this observation may help to develop advanced protocols for stem cell-based therapy approaches in regenerative medicine.

## 4. Materials and Methods

### 4.1. Animals

Mice were maintained at the animal facility of the Max-Delbruck Center, in pathogen-free conditions, in individually ventilated cages, in accordance with the German Animal Protection Law. *Tph1^−^*^/*−*^ [1] and *Tph2^−^*^/*−*^ [14] mice, on the C57Bl/6 background, were cross-bred to generate *Tph1/Tph2^−^*^/*−*^ mice [40].

### 4.2. Cells

All cell culture reagents, when not specified, were purchased from Thermo Fisher Scientific, Waltham, MA, USA. Murine embryonic fibroblasts (MEFs) were isolated from 13.5–14.5 dpc wild-type (WT) and *Tph−*deficient mouse embryos [14], and grown in MEF medium consisting of high glucose DMEM medium, 10% FBS, 100 U/mL penicillin, 100 mg/mL streptomycin, 2 mM L-Glutamine. Mouse iPSCs were routinely cultured in mouse embryonic stem (MES) medium: knockout DMEM media, 15% FBS (Sigma-Aldrich, St. Louis, MO, USA), 100 U/mL penicillin, 100 mg/mL streptomycin, 2 mM L-Glutamine, 1 μM non-essential amino acids NEAA, 50 μM β-mercaptoethanol (β-MET, Sigma-Aldrich, St. Louis, MO, USA), in-house produced leukemia inhibitor factor LIF (1:5000). Reprogramming was run in N2B27 2i media, consisting of standard N2B27 medium (1/2 Neurobasal supplemented with 1 × N2, 1/2 DMEM/F12, 1 × B27 component, 100 U/mL penicillin, 100 mg/mL streptomycin, 2 mM L-Glutamine, 25 μM β-MET (Sigma-Aldrich, St. Louis, MO, USA)) supplemented with 3 μM CHIR-99021, 1 μM PD-0325901 (Axon Medchem, Groningen, the Netherlands), recombinant in-house made hLIF (5 ng/mL), and 3 μg/mL Dox (Sigma-Aldrich, St. Louis, MO, USA). All cells were cultured at 37 °C in a standard CO_2_ incubator.

### 4.3. Lentiviral Vectors

The murine *Tph1* (NM_009414.3) and *Tph2* cDNAs (NM_173391.3) were RT-PCR amplified from total mRNA of mouse intestine and brain tissues, accordingly, with the use of primers for *Tph1*: Tph1-AscI-F 5’-TATGGCGCGCCCATGATTG-AAGACAACAAGGAGAAC-3’ and Tph1-BsaI-R 5’-CACGGTCTCACTAGTGAAACCA-TCACACACTGGGC-3’; and for *Tph2*: Tph2-AscI-F 5’-TATGGCGCGCCCATGCAGCCCGCAATGATG-3’ and Tph2-SpeI-R 5’-GCCCGGCACTAGTGGCATCAAATCCCCAGATATTGGT-3’. The DNA fragments were cloned into pLVTHM-T7-hnRNP-K(iso1/2)-iresPuro plasmid digested with AscI and SpeI (New England Biolabs, MA, USA) restriction enzymes, replacing the hnRNP-K insert [66]. For *Tph1* cloning, the SpeI site of the reverse primer was replaced with BsaI, mimicking SpeI sticky end. The resulting plasmids—pLVTHM-T7-Tph1-iresPuro and pLVTHM-T7-Tph2-iresPuro—were used for TPH1 and TPH2 overexpression, respectively. The pLVTHM-T7-Empty-iresPuro control plasmid was derived from pLVTHM-T7-Tph2-iresPuro, digested with AscI and SpeI (New England Biolabs, Ipswich, MA, USA) restriction enzymes, followed by blunting and re-ligation.

### 4.4. Lentivirus Packaging

For lentivirus packaging, HEK293T cells were transiently co-transfected with envelope (pMD2G), packaging (psPAX2) plasmids, and different target plasmids: pLVTHM-T7-Tph1-iresPuro, or the same vector containing Tph2 or empty control plasmid, or M2rtTA plasmid or pHAGE2-OKSM plasmid, carrying reprogramming factors (Oct4, Klf4, Sox2, cMyc) [67]. Lentiviruses from cell culture supernatants were collected and concentrated as described elsewhere [68,69]. Titers for the lentiviral stocks were around 10 − 20 × 10^6^ TU/mL.

### 4.5. Reprogramming MEFs into iPSCs

Cell reprogramming was performed as described [70]. MEFs (2 lines of each genotype) from WT, *Tph1*^−/−^, *Tph2*^−/−^ [14], and double knockout *Tph1/Tph2*^−/−^ [40] mice (all lines on the C57BL/6 genetic background) were grown in standard MEF media in a CO_2_-incubator: 5% CO_2_ at 37 °C. Cells were seeded (3 × 10^4^ cells per well) on a 0.1% gelatin-coated 24-well plate in the MEF medium. Next day, media were replaced with 200 μL of Opti-MEM media (Gibco), containing a mixture of lentiviruses (MOI = 2–5 for each): M2rtTA + pHAGE2-OKSM [67]. Cells were incubated with the virus mixtures for 3–4 h, then 200 μL of Opti-MEM was added and incubation was continued overnight. Next day, the media were changed to MEF media, containing 3 μg/mL Dox, and supplemented with chemicals corresponding to the particular experiment: 50 nM serotonin (Sigma-Aldrich, St. Louis, MO, USA), or 10 or 40 μM ZDON (#616467, Merck, Darmstadt, Germany). The media were changed to fresh every day, and on the 3rd day, cells were trypsinized and seeded onto 3 wells of 12- or 6-well plates, with pre-plated mitomycin C-treated MEFs, and then cultured in N2B27 2i media at 37 °C, in 5% CO_2_. Medium was changed once per 1–2 days. Clones became visible on day 9; on day 14–15 they were picked or fixed/proceeded for immunostaining. To ensure reproducibility, all reprogramming experiments were repeated twice.

### 4.6. TPH Overexpression and Rescue Experiments during iPSC Generation

WT or *Tph1/Tph2*^−/−^ MEFs were seeded onto 0.1% gelatin-coated wells of 6-well plates (25 × 10^3^ cells per well), and infected next day (as described above) with the pLVTHM-T7-Tph1-iresPuro, pLVTHM-T7-Tph2-iresPuro (each at MOI = 2), and “empty” pLVTHM-T7-iresPuro (as control) lentiviral particles. The total amount of T7-contaning lentiviruses was equal in each well. For the transduction with Tph1 or Tph2 alone, the TU per well was adjusted, by adding lentiviruses harboring “empty” pLVTHM-T7-iresPuro vector. After 24 h, cells were grown for 4 days in MEF medium containing puromycin (1 μg/mL), and for 2 more days in standard MEF medium. Thereafter, cells were trypsinized and seeded onto MEF feeder-containing 6-well plates (with cell density 1 × 10^5^ per well), and proceeded to reprogramming with M2rtTA/pHAGE2-OKSM (as described above) [70,71]. After immunostaining (see below), Nanog and T7 tag-positive iPSC colonies were analyzed in four technical replicates (*n* = 4 in Section 2.2) for each experimental genotype per well.

### 4.7. Immunostaining

Immunostaining was performed as previously described [72]. Mouse iPSCs colonies (day 12–17 of reprogramming) were fixed with 4% paraformaldehyde (PFA, Sigma-Aldrich, St. Louis, MO, USA), washed with PBS, permeabilized with 0.1% Triton-X100 in PBS (PBST), and treated for 30 min with blocking solution (each step was accompanied with PBST washing): 1% BSA, 2% non-immune sheep serum, 0.1% Tween-20 in PBS (Thermo Fisher Scientific, Waltham, MA, USA). Fixed cells were incubated with antibodies against mouse Nanog (Bethyl Labs, Montgomery, TX, USA), TPH (T0678, Sigma-Aldrich, St. Louis, MO, USA), T7 tag (Thermo Fisher Scientific, Waltham, MA, USA), followed by staining with Cy3 or Alexa-488-labeled secondary antibodies (Jackson ImmunoResearch, West Grove, PA, USA). Immunocytochemistry results were visualized using the EVOS™ FL Auto Imaging System (Thermo Fisher Scientific, Waltham, MA, USA).

### 4.8. Alkaline Phosphatase Staining

Mouse iPSC colonies were fixed with PFA and stained for alkaline phosphatase (AP), as described in [70], with some minor modifications. PFA-fixed clones were washed in PBS and 25 mM Tris-maleate buffer (pH 9.0), and then incubated with the substrate mixture containing Tris-maleate buffer (pH 9.0), 4 mM MgCl_2_, 0.2 μg/mL 1-Naphthyl phosphate disodium salt, and 0.5 μg/mL Fast Red TR salt (Sigma-Aldrich, St. Louis, MO, USA), or SigmaFast Fast Red TR/Naphthol AS-MX tablets (Sigma-Aldrich, St. Louis, MO, USA). The reaction was stopped by washing with PBS.

### 4.9. Immunoblotting

Immunoblotting was performed as previously described [73]. Mouse iPSCs pellets were resuspended with Laemmli loading buffer. After 10% SDS-PAGE electrophoresis, and the protein transfer to the nitrocellulose membrane (Bio-Rad, Hercules, CA, USA) with semi-dry transfer (Helikon, Russia), the TPH and beta-actin (a loading control) proteins were detected with mouse monoclonal antibodies T0678 (Sigma-Aldrich, St. Louis, MO, USA) and MA1-744 (Thermo Fisher Scientific), respectively. Anti-mouse IgG HRP-conjugated antibodies were used as secondary antibodies (#115-036-062 and #111-036-045, Jackson ImmunoResearch, USA). Chemiluminescence was detected with a ChemiDoc Touch Imaging System (Bio-Rad, Hercules, CA, USA).

### 4.10. Teratoma Formation and Histological Analysis

Teratoma analysis was performed as described previously [72]. Briefly: iPSCs were trypsinized, washed in PBS, and 10^6^ cells were subcutaneously injected in the hindlimb of athymic NUDE mice (*Crl:NU(NCr)-Foxn1nu*). After 3–4 weeks, the mice were euthanized, and teratomas were removed and fixed in 4% paraformaldehyde in PBS. Teratomas were cut into pieces with a 5 mm diameter, dehydrated, and paraffinized. The paraffin pieces were then cut into 5 μm slices using a microtome Leica RM2235 (Leica Biosystems, Wetzlar, Germany). The glass-slide attached paraffin slices were dried, rehydrated, and stained with hematoxylin, washed and then stained with eosin. Then they were washed, dehydrated, and mounted with Canadian balsam and coverslip. The teratoma histological sections were analyzed using the EVOS Cell Imaging Systems (Thermo Fisher Scientific, Waltham, MA, USA).

### 4.11. HPLC

HPLC measurements of serotonin, and its metabolite, 5-hydroxyindoleacetic acid (5-HIAA), were performed using HPLC with electrochemical detection (Eicom, HTEC-500, Sapporo, Japan), with a carbon electrode WE-3G (Eicom, Sapporo, Japan), using +650 mV applied potential, and equipped with a reverse-phase column CA-50DS (150 × 2.1 mm, Eicom, Sapporo, Japan), at a flow rate of 200 μL/min. Cells in logarithmic growth phase were trypsinized, washed 2 times with PBS, and immediately frozen and stored at −80 °C in a refrigerator. The cell samples were homogenized in 0.1 M HClO4, centrifuged (10 min, +4 °C; 14,000× *g*), and filtered using centrifuge filter units (PVDF membrane, pore size 0.22 μm, Millipore, Burlington, MA, USA). The mobile phase contained 100 mM PBS, 0.17 mM disodium ethylenediaminetetraacetate (EDTA), 1.8 mM octane sulfonic acid sodium salt, and 18% (vol/vol) methanol, pH 4.5. All peaks obtained were normalized to the internal standard 3,4-dihydroxybenzylamine, and final values for serotonin and 5-HIAA were counted as ng/mL cell extract of equal number of cells.

### 4.12. Statistical Analysis

Statistical significances of observed differences were evaluated by two-tailed *t*-tests.

## Data Availability

The datasets used and/or analyzed during the current study are available from the corresponding author on reasonable request.

## Supplementary Materials

The following supporting information can be downloaded at: https://www.mdpi.com/article/10.3390/ijms24054862/s1.
