# Supplementary material for: Tryptophan Hydroxylase-2-Mediated Serotonin Biosynthesis Suppresses Cell Reprogramming into Pluripotent State
Source: Int J Mol Sci. 2023 Mar 2;24(5):4862. doi: 10.3390/ijms24054862 (PMC10003565; doi:10.3390/ijms24054862)
Supplement: Supplementary file 1 [file ijms-24-04862-s001.zip › ijms-2211180-supplementary.pdf]

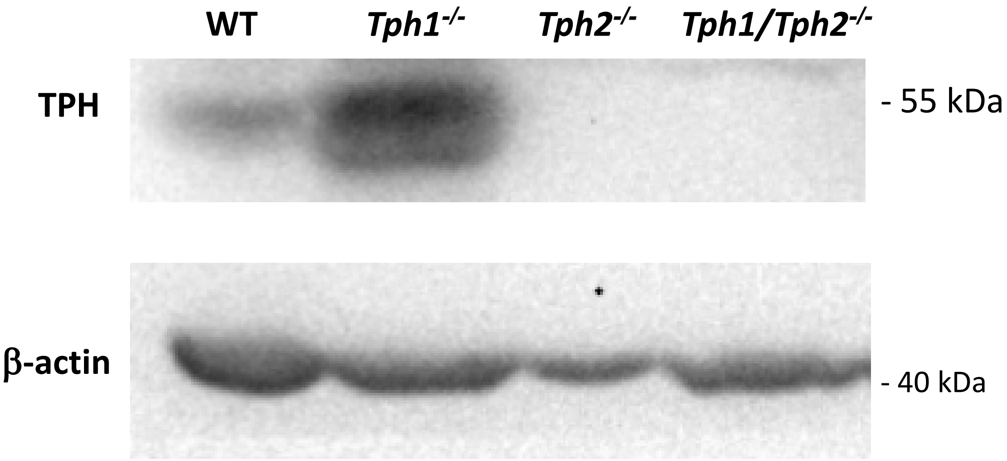

Supplementary Figure S1. ***Tph2*** $^{-/-}$ **and** ***Tph1/Tph2*** $^{-/-}$ **knockout iPSCs do not express TPH proteins.** ***Tph1*** $^{-/-}$ **iPSCs express excess of TPH2.** Representative immunoblotting of iPSCs lysates from WT and *Tph*-knockouts with pan-TPH antibodies and beta-actin antibodies as loading control.

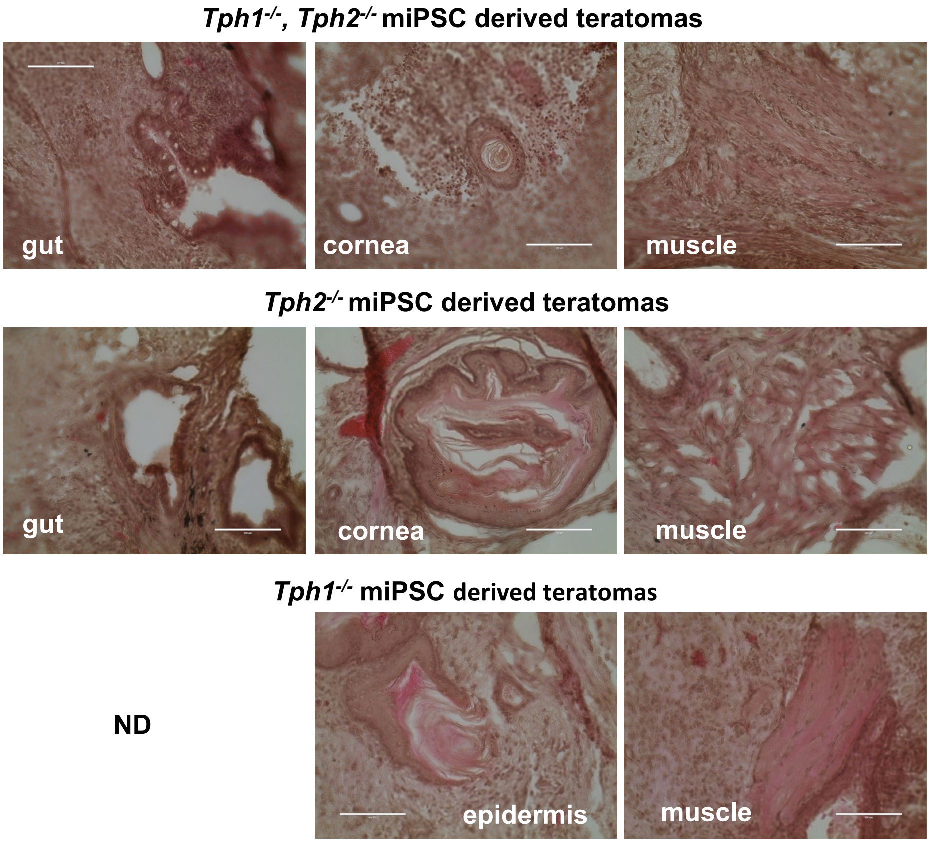

Supplementary Figure 2. iPSCs lacking TPH enzymes develop teratomas with all three embryonic germ layers. *Tph1/Tph2*$^{-/-}$ and *Tph2*$^{-/-}$ teratomas showed development of gut, cornea and muscle tissues representing endoderm, ectoderm and mesoderm germ layers, respectively. In *Tph1*$^{-/-}$ iPSC-derived teratomas the ectodermal tissues were not determined (ND).

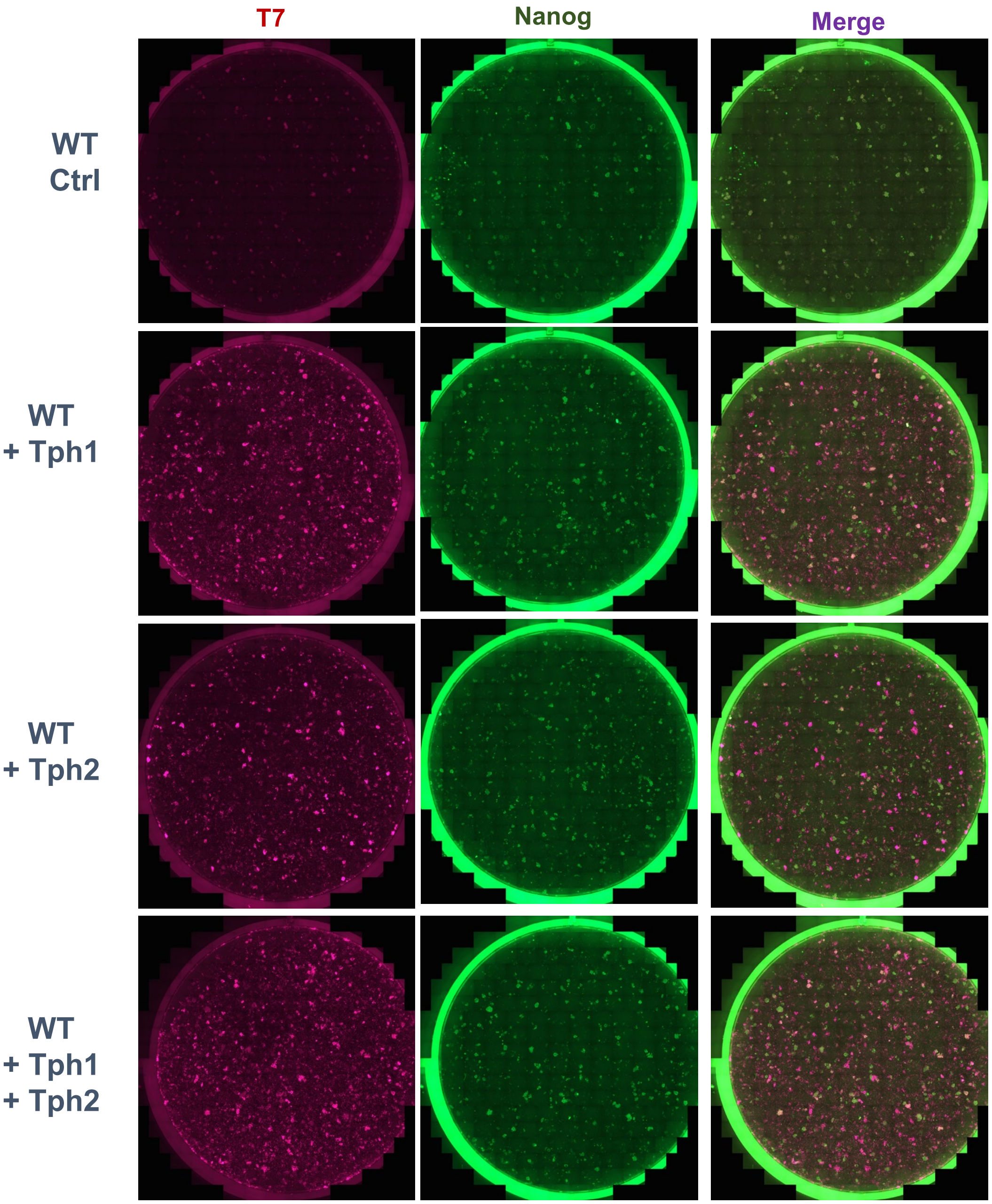

Supplementary Figure 3. Overexpression of Tph1 и Tph2 during OKSM reprogramming improves the efficiency of iPSC generation. Representative immunostainings of WT iPSCs clones after 14 days of OKSM-reprogramming in conditions of continuous overexpression of Tph1 or Tph2 or both with anti- T7-epitope and -Nanog antibodies.

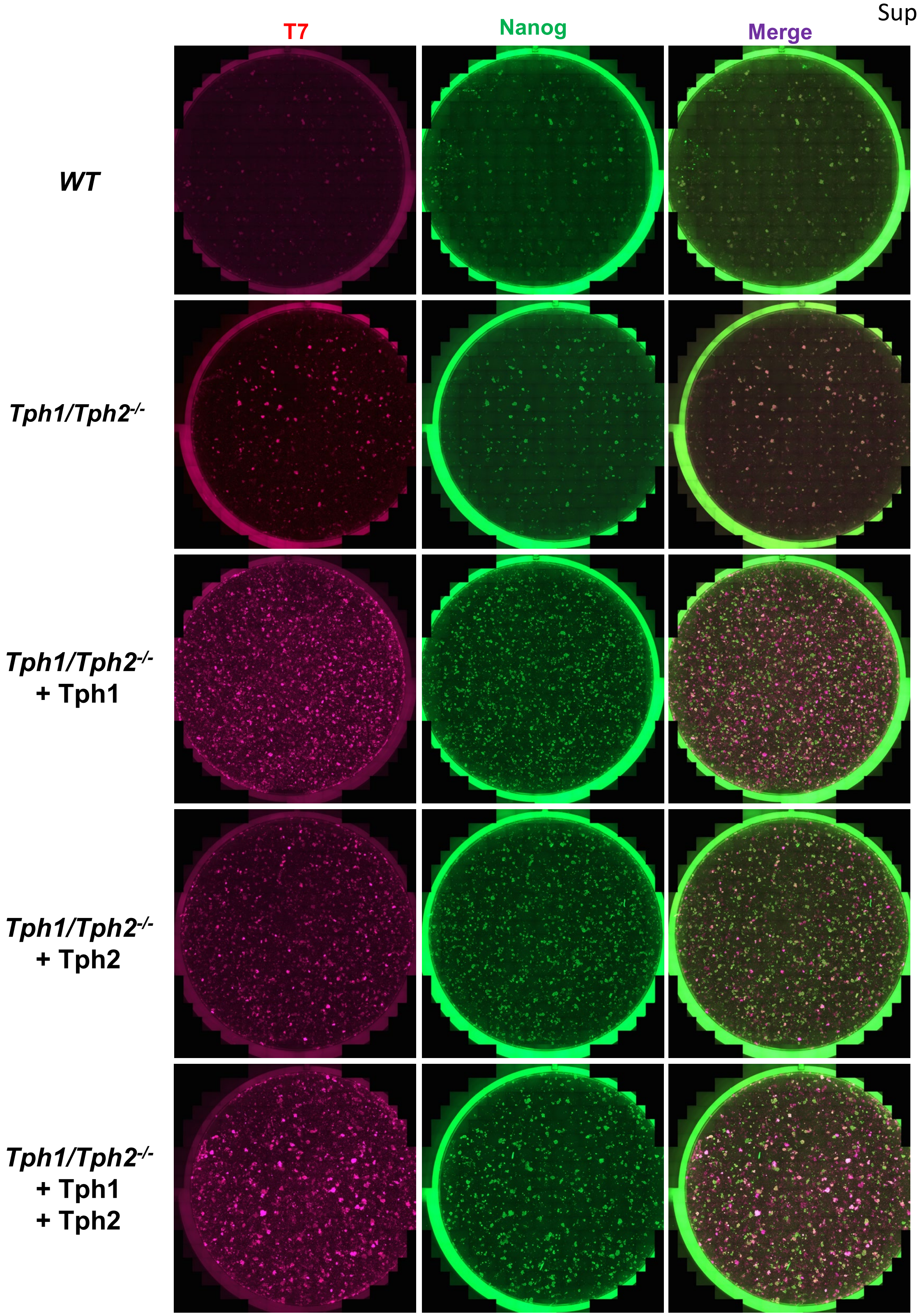

Supplementary Figure 4. Tph2 overexpression rescues the *Tph1/Tph2$^{-/-}$* phenotype. Representative immunostainings of *Tph1/Tph2$^{-/-}$* iPSC clones after 14 days of OKSM-reprogramming in conditions of continuous overexpression of Tph1 or Tph2 or both with anti-T7-epitope and Nanog antibodies.

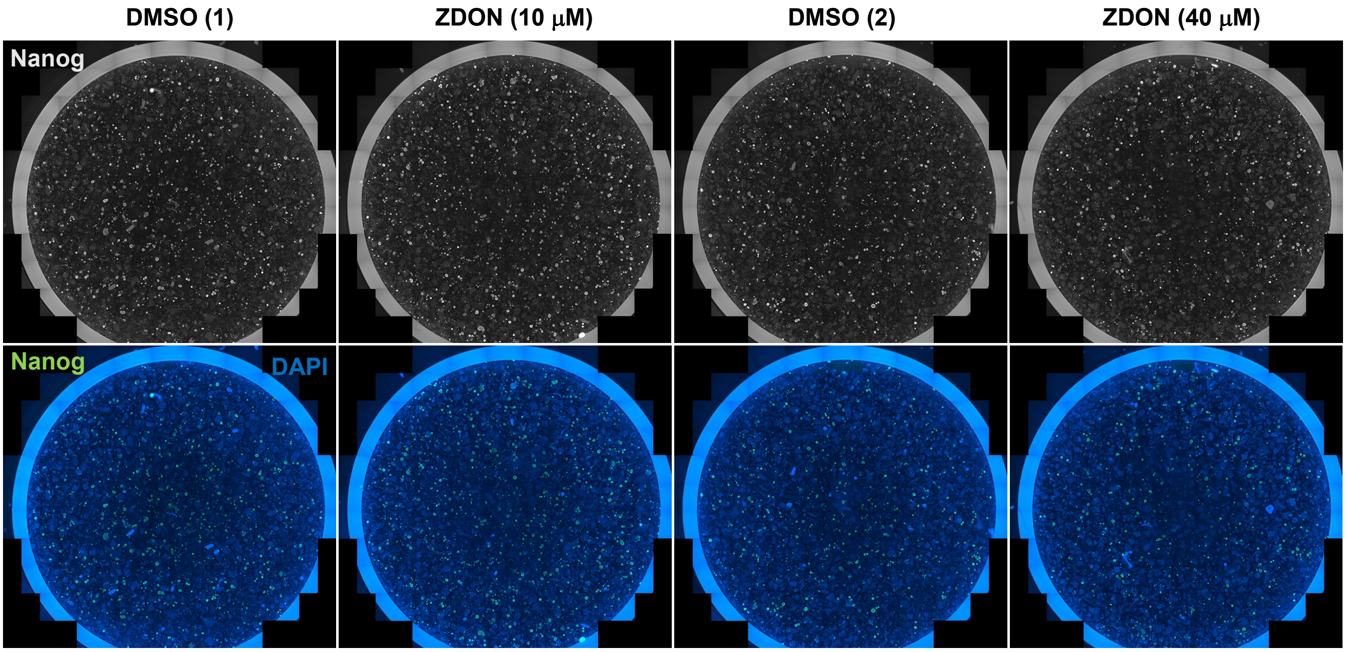

Supplementary Figure 5. Inhibiting serotonylation by 10 μM ZDON does not affect the reprogramming efficiency of WT MEFs. Representative immunostainings of WT iPSCs clones after 14 days of OKSM-reprogramming and continuous treatment with ZDON (10 and 40 μM) with anti-Nanog antibodies.

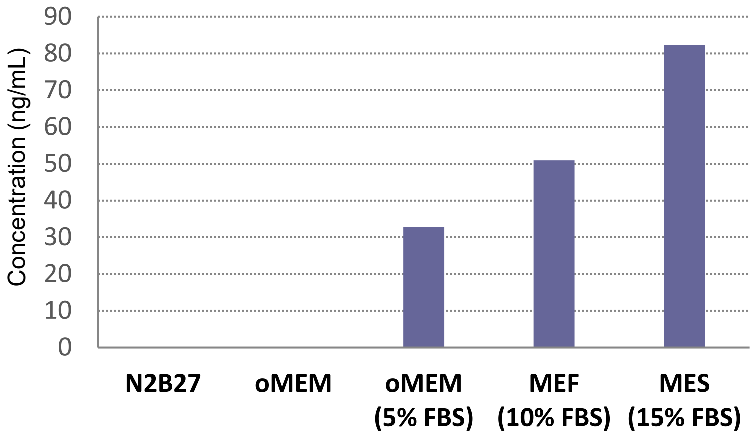

Supplementary Figure 6. Serotonin content in the cell culture media depends on the serum concentration. HPLC-based detection of serotonin in different culture media used for cell reprogramming.

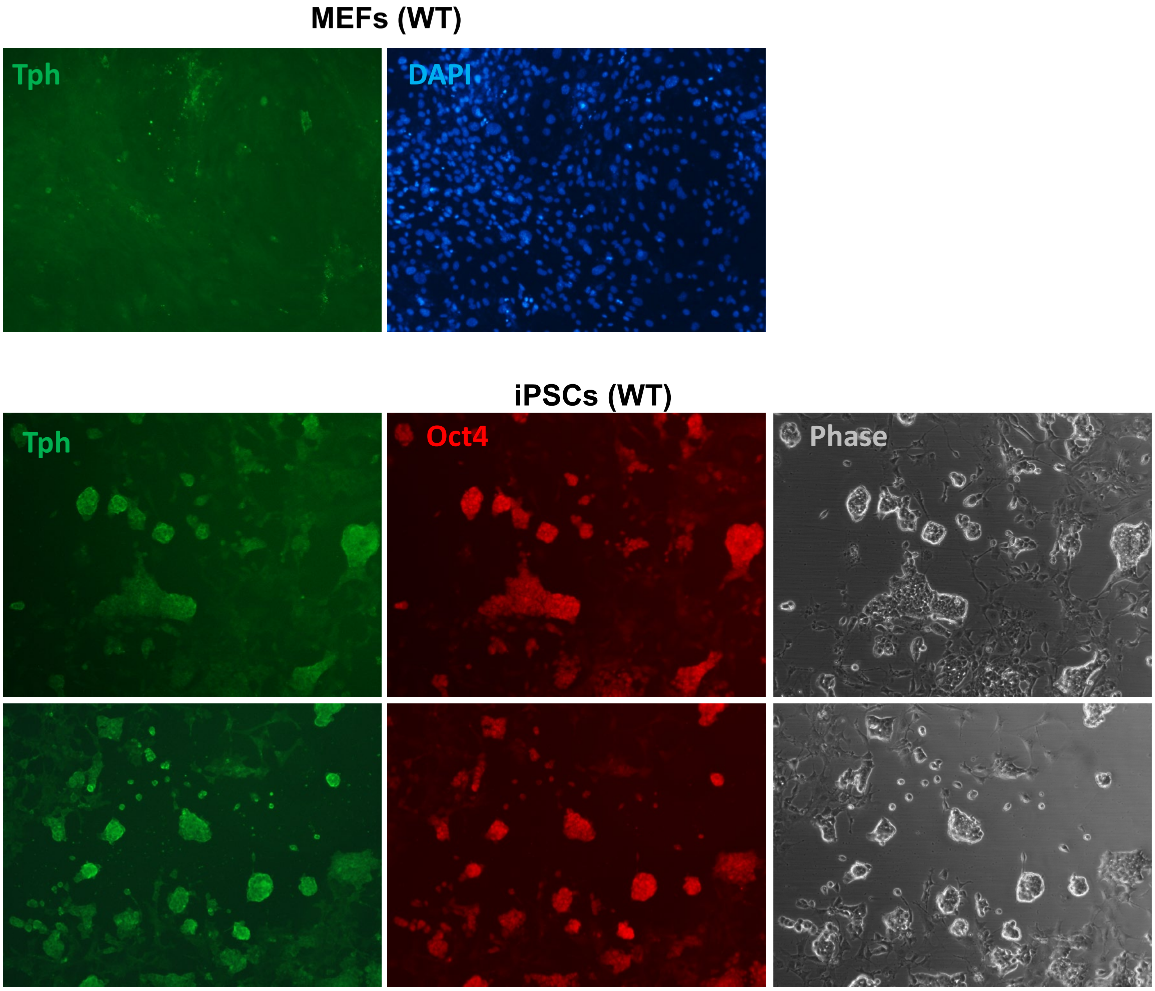

Supplementary Figure 7. TPH is highly expressed in Oct4-positive iPSC clones and in rare MEFs. Representative immunostaining of WT MEFs and iPSCs with anti-pan-TPH and Oct4 antibodies.
